# Coupled immune stratification and identification of therapeutic candidates in patients with lung adenocarcinoma

**DOI:** 10.18632/aging.103775

**Published:** 2020-08-27

**Authors:** Weilei Hu, Guosheng Wang, Yundi Chen, Lonny B. Yarmus, Biao Liu, Yuan Wan

**Affiliations:** 1 Institute of Translational Medicine, Zhejiang University, Hangzhou 310029, China; 2 Center for Disease Prevention Research and Department of Pharmacology and Toxicology, Medical College of Wisconsin, Milwaukee, WI 53226, United States; 3 The Pq Laboratory of Micro/Nano BiomeDx, Department of Biomedical Engineering, Binghamton University—SUNY, Binghamton, NY 13902, United States; 4 Division of Pulmonary and Critical Care, Department of Medicine, Johns Hopkins School of Medicine, Baltimore, MD 21218, United States; 5 Department of Pathology, Nanjing Medical University Affiliated Suzhou Hospital, Suzhou 215006, Jiangsu, China

**Keywords:** personalized cancer immunotherapies, drug repositioning, cold tumor, patient stratification, tumor microenvironment

## Abstract

In recent years, personalized cancer immunotherapy, especially stratification-driven precision treatments have gained significant traction. However, due to the heterogeneity in clinical cohorts, the uncombined analysis of stratification/therapeutics may lead to confusion in determining ideal therapeutic options. We report that the coupled immune stratification and drug repurposing could facilitate identification of therapeutic candidates in patients with lung adenocarcinoma (LUAD). First, we categorized the patients into four groups based on immune gene profiling, associated with distinct molecular characteristics and clinical outcomes. Then, the weighted gene co-expression network analysis (WGCNA) algorithm was used to identify co-expression modules of each groups. We focused on C3 group which is characterized by low immune infiltration (cold tumor) and wild-type EGFR, posing a significant challenge for treatment of LUAD. Five drug candidates against the C3 status were identified which have potential dual functions to correct aberrant immune microenvironment and also halt tumorigenesis. Furthermore, their steady binding affinity against the targets was verified through molecular docking analysis. In sum, our findings suggest that such coupled analysis could be a promising methodology for identification and exploration of therapeutic candidates in the practice of personalized immunotherapy.

## INTRODUCTION

Current understanding of cancer immunology has promoted the stratification of patients for identifying and exploring new cancer immunotherapeutic strategies [1, 2]. Immunohistochemical staining-based immunoscore system is a possible approach in the classification of malignant tumors [3–5]. For example, lymphocyte infiltration and high expression level of IFN-γ (T cell-inflamed tumors, *i.e.*, hot tumors) may segregate tumors, indicate patients may benefit from PD-1/PD-L1 inhibitors, and help predict immunotherapy responsiveness [6, 7]. On the contrary, the non-T cell-inflamed phenotype, *i.e.*, cold tumors, lacks expression of the type I IFN signature, CD8+ T cells, and IFN-inducible inhibitory factors, correlated with treatment resistance. In addition, bulk gene expression profiling methods, such as CIBERSORT, TIMER, and integrated immunogenomic methods [8–13] have also been developed to characterize the immune landscape of cancer and to help guide cancer immunotherapy. However, these stratification approaches are mainly limited by heterogeneity in clinical cohorts, probably leading to confusion in determining ideal therapeutic options. Theoretically, the limitations can be partially offset by coupled analysis of stratification/therapeutics, which is relatively straightforward and efficient. However, no attempt has been undertaken.

**Figure 1:**
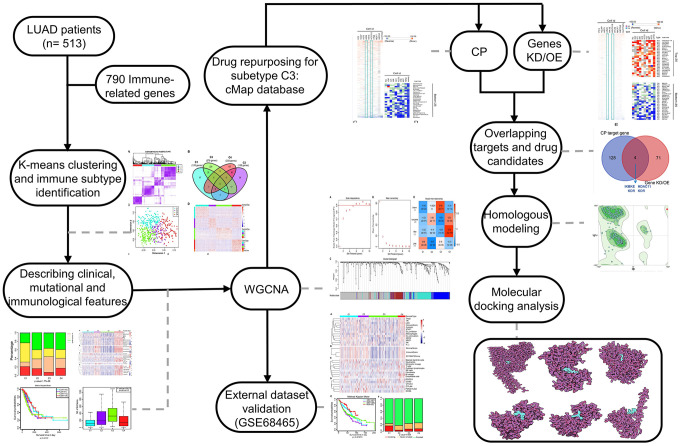
**The workflow of the study.** CMap, connectivity Map; WGCNA, Weighted correlation network analysis; CP, compound; Genes KD/OE, genes knockdown/overexpress.

Drug repurposing is a strategy for identifying new uses for approved or investigational drugs that are outside the scope of the original medical indication [14, 15]. Compared to *de novo* drug discovery, drug repurposing can significantly reduce the cost and time to bring a new treatment to patients. It is possible now to link gene-expression profiling data and screens for drug repurposing [16, 17]. Moreover, the Connectivity Map (CMap) database, based on a computational drug repurposing approach, has been demonstrated as an efficient tool for drug repurposing [18–20]. Therefore, combining genome polymorphisms and pharmacology may lead to promising new therapeutic strategies [21], and several drugs have been repurposed to treat cancers [22–24]. Of note, a careful selection of pertinent groups for evaluation of drug candidates remains essential, which reversely requires the rational stratification before drug repurposing. In this work, patient stratification and drug repurposing were coupled to explore novel therapeutic candidates for treatment of LUAD which accompanied with marked genetic and genomic heterogeneity [25, 26]. Following the steps shown in Figure 1, we categorized the patients into four groups based on immune gene profiling and then identified five drugs targeting four known targets with a computational drug repurposing approach. These identified agents could correct aberrant gene expression in a class of patients referred to as the C3 group, which is characterized by cold tumors and expression of wild-type EGFR. The binding affinity between these potential drugs and paired targets were also investigated with molecular docking methods.

## RESULTS

### Four LUAD subtypes were delineated based on the immune-associated genes

**Figure 2:**
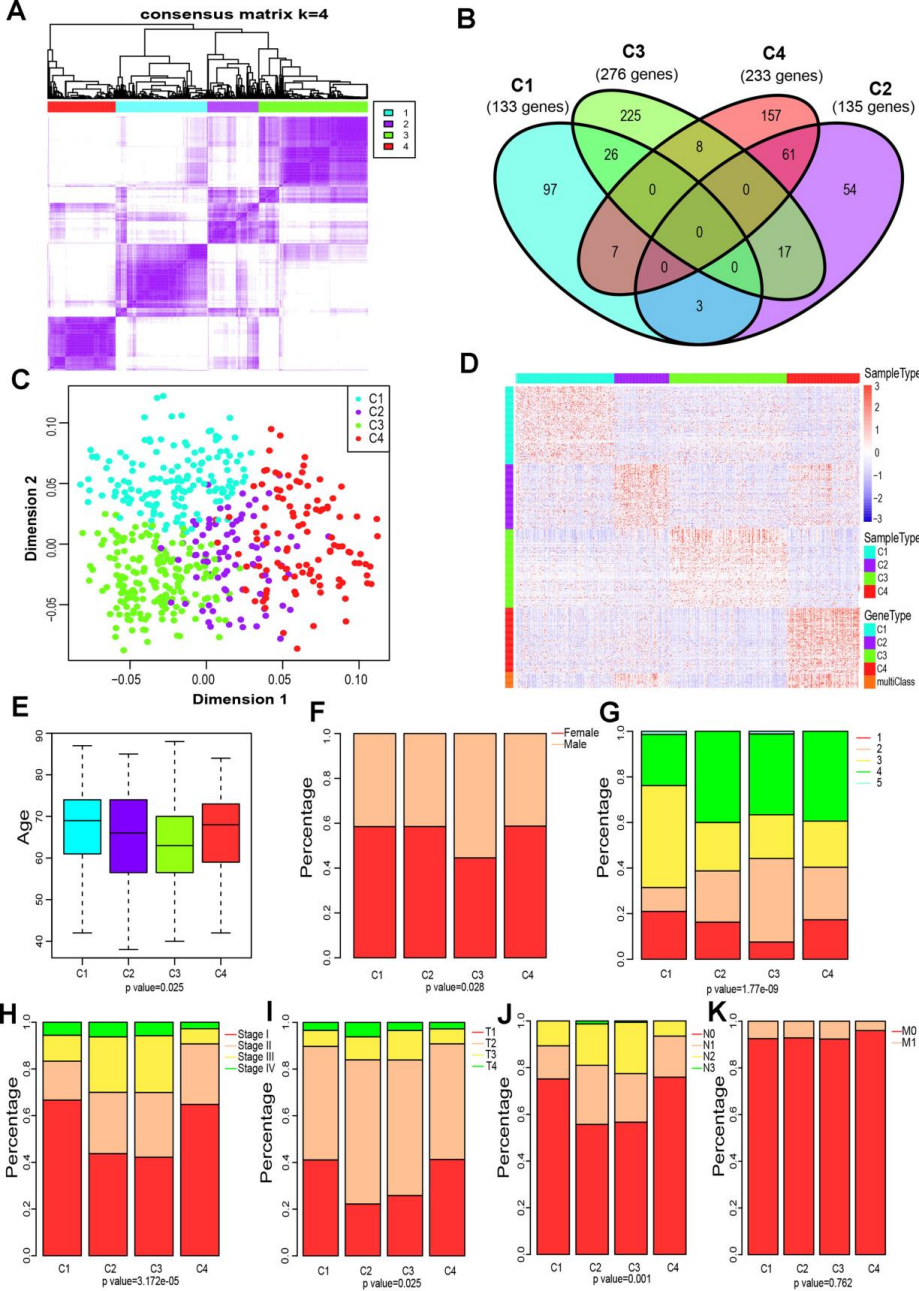
**Four immune subtypes of LUAD in TCGA cohort and their clinical profiles.** (**A**) Heatmap of consensus values when k=4. (**B**) Venn diagram showing the upregulated genes (FDR < 0.05) in each cluster. (**C**) The scatter plot of the top 100 upregulated genes in each cluster, distinguished by the first two principal components (PCs). (**D**) Gene expression profile of the top 100 upregulated genes in each cluster. Heat maps showing relative gene expression values, red indicates high expression, and blue indicates low expression. (**E**) Age at diagnosis of the four subtypes (Kruskal-Wallis test). The Boxplot centerlines indicating the median value; box limits show the 25^th^ (Q1) and 75^th^ (Q3) percentiles, lower and upper whiskers extend 1.5 times the interquartile range (IQR) from Q1 and Q3, respectively. (**F**) Distribution of gender among the four subtypes (chi-square test). (**G**) Distribution of smoking status across the four subtypes (chi-square test). (**H**) Distribution of stage at diagnosis in the four subtypes (chi-square test). (**I**) The degree of progression of the primary tumor (T) at diagnosis in the four subtypes (chi-square test). (**J**) The degree of the invasion of regional lymph nodes (N) at diagnosis among the four subtypes (chi-square test). (**K**) Incidence of metastatic (M) dissemination at diagnosis among the four subtypes (chi-square test).

The gene expression profiles of 790 immune-associated genes were used to classify the TCGA cohort data into different LUAD subtypes. Initially, we assigned all tumor specimens into k (k = 2, 3, 4, 5, 6, 7, 8) subtypes. A value of k = 4 was set to represent stable clusters according to the CDF curves of the consensus score (Figure 2A and Supplementary Figure 1). A total of 513 LUAD tumor samples were finally assigned to four categories. The Kolmogorov-Smirnov test was used to calculate the upregulated genes in each subtype (FDR < 0.05). Of the 790 immune-associated genes, 133, 135, 276, and 233 genes were remarkably enriched in subtypes C1, C2, C3, and C4, respectively (Figure 2B). It is worth noting that only a few genes overlapped between pairs of subsets (Figure 2B). Next, principal component analysis (PCA) was employed to calculate the top 100 highly expressed genes in each cluster. The four subsets were distinguished from each other based on the two-dimensional scaling plotting of the first two principal components (Figure 2C). Furthermore, the top 100 enriched genes in each subtype were used to describe their immune gene expression profiles (Figure 2D). In addition, the R package sigclust was utilized to analyze the clustering significance between the four consensus clusters. It was found that the comparison between C2 and C3 was not significant (p=0.192), but marked differences were observed in expression distribution of C1 vs C4, C3 vs C4 (p < 0.05) (Supplementary Table 1). Therefore, the 513 LUAD patients extracted from TCGA cohort were classified into five molecular subtypes depending on the expression pattern of immune-associated genes.

### Clinical profile of the four subtypes

To investigate the clinical relevance of tumor immune microenvironment, demographic distributions of age, gender, smoking status, tumor stage and the degree of progression of the primary tumor (T), tumor cells invasion into regional lymph nodes (N) and metastatic dissemination (M) were compared between patients with the four immune subtypes. Clinically, we observed that C3 subtype have a markedly lower median age at diagnosis (p = 0.025 Pearson’s chi-square test, Figure 2E), and the highest proportion of male patients (p=0.028 Pearson’s chi-square test, Figure 2F) and smokers (1.77 × 10^−9^ Pearson’s chi-square test, Figure 2G). Groups C2 and C3 showed a similar frequency of Stage II, Stage III and Stage IV, which is significantly higher than that of group C3 or C4 (p=3.172 × 10^−5^ Pearson’s chi-square test, Figure 2H). Specifically, groups C2 and C3 displayed a higher proportion of T3 and T4 (p=0.025 Pearson’s chi-square test, Figure 2I), and a much lower percentage of N0 (p=0.001 Pearson’s chi-square test, Figure 2J) compared to C1 or C4. Interestingly, the metastatic dissemination rate at diagnosis was not different among the four groups (p =0.762 Pearson’s chi-square test, Figure 2K).

### Distinct characteristics of immunogenicity of the LUAD subtypes

**Figure 3:**
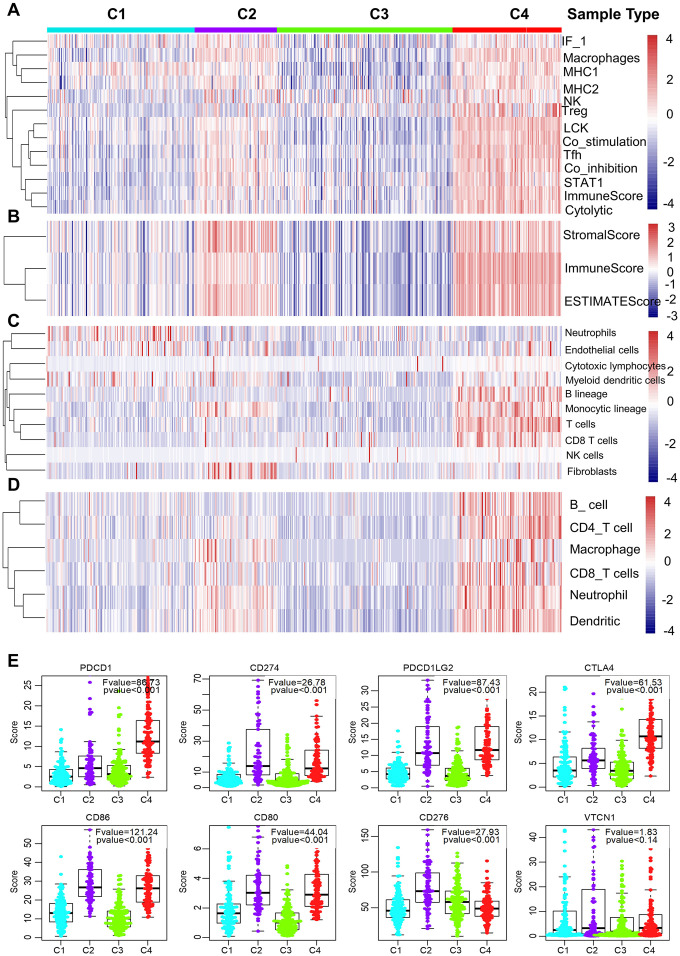
**Immune signature of the four subtypes in the TCGA cohort.** (**A**–**D**) Heatmaps showing the gene expression scores of immune profiles of the four subtypes. A two-color scale was used, with red indicating high expression and blue representing low expression. (**A**) The expression levels of 13 immune metagenes among the four subtypes. The 13 immune metagenes: IF1, macrophages, MHC2, MHC1, NK, T regulatory cells, lymphocyte-specific kinase (LCK), STAT1, T follicular cells, T cell inhibitory and stimulatory activity, and immune score and cytolytic activity. (**B**) The expression scores of genes included in the ESTIMATE algorithm for determination of stromal and immune gene signatures. (**C**) The expression scores of 10 groups of immune-associated cells. (**D**) The expression levels of genes included in the TIMER algorithm for assessment of immune infiltrates. (**E**) Differential expression of checkpoint molecules among the four immune subtypes. Boxplots indicate 5%, 25%, 50%, 75%, and 95%, respectively. Comparisons between subtypes were performed by Analysis of Variance (ANOVA). P-values were corrected by the Bonferroni method.

We further examined the immunogenic and microenvironmental variables including immune cell metagene expression level, immune cells, tumor purity, immune and stromal score, and the abundance of tumor-infiltrating lymphocytes using RNA expression data as previously described [27]. All immunogenic and microenvironmental factors scores were considerably lower in subtype C3 compared to C4. Immune metagenes corresponding to macrophages, NK, Treg, Tfh and LCK cells, and expression of co-stimulation/co-inhibition signal-associated genes, MHC class I/II, interferon and interferon regulated genes (STAT1) were markedly lower in subtype C3 than in C4 (Figure 3A and Supplementary Figure 2). In terms of tumor microenvironment factors (stomal score, immune score, tumor purity), subtypes C2 and C4 showed upregulated stromal and immune genes and estimated tumor purity, while the subtype C3 tumors exhibited low levels of immune and stromal genes and estimated tumor cell fraction (Figure 3B and Supplementary Figure 2).

Additionally, a higher number of immune-associated cells such as, B lineage cells, monocytic lineage cells, T cells, and CD8 T cells were produced in subtype C4 than in other subtypes, while endothelial cells and myeloid dendritic cells responded more aggressively to subtype C3 (Figure 3C and Supplementary Figure 2). Molecular-tumor interactions were comprehensively assessed with TIMER (https://cistrome.shinyapps.io/timer/). Similarly, we compared the number immune infiltrating cells (dendritic cells, neutrophils, B, CD8^+^ T, CD4^+^ T, macrophages) in TCGA LUAD samples. We found that these immune cells were fewer in subtype C3 than in C4 (Figure 3D and Supplementary Figure 2). It was also noted that there was significant immune infiltration and higher expression of immune-associated genes in subtype 4, showing an enhanced immune microenvironment and disrupted immune microenvironment in subtype C3.

The expression profiles of eight immune checkpoint genes, which are crucial for immune modulation, were further examined (Figure 3E). The following genes were considerably lower in subtype C3 compared to C4, i.e., PDCD1 (PD1), CTLA4, CD274 (PDL1), PDCD1LG2 (PDL2), CD80 and CD86. Interestingly, the expression value of CD276 was markedly downregulated in subtype C4 whereas the expression level of VTCN1 was similar among the four subtypes.

### Prognostic values of the four LUAD subtypes

**Figure 4:**
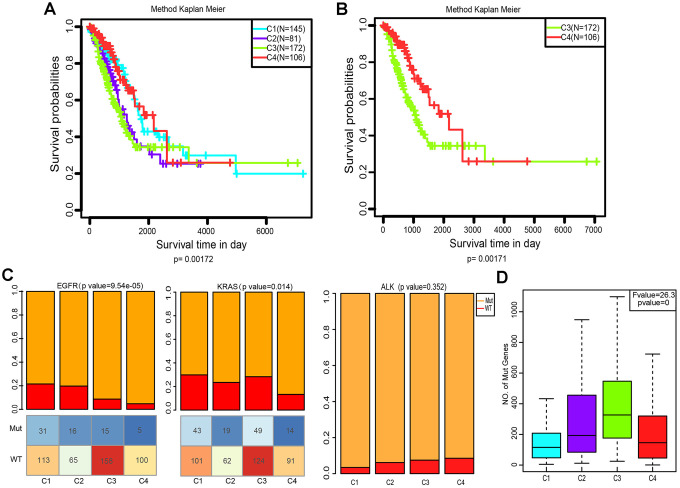
**Kaplan–Meier curves and mutation status of the four immune subtypes**. (**A**) Overall survival (OS) of the four subtypes (log-rank test). (**B**) Five-year Kaplan–Meier curves for OS of subtypes C3 and C4 (log-rank test). (**C**) Distribution of EGFR, KRAS and ALK mutant among the four subtypes. The lower half represents the number of EGFR/KRAS mutant and wild-type of different subtypes (chi-square test). (**D**) Distribution of the number of mutant genes in the four samples (Analysis of Variance, p<0. 0001).

We then explored whether the immune-associated genes can predict the prognosis of patients with the four subtypes. The Kaplan-Meier curves were plotted to reveal the overall survival (OS) rates of patients (log-rank test, OS, p=0.00172, Figure 4A). Notably, C4 had the highest OS rate among the four subtypes. In comparison, patients with subtype C3 had a worse OS than those in other subtypes, especially in C4 (log-rank test, OS, p=0.00172, Figure 4A; log-rank test, OS, p=0.00171, Figure 4B).

### Comparison of EGFR, KRAS and ALK mutations among the four subtypes

Aberrant changes in KRAS, EGFR, ALK have been recognized as key drivers of lung cancer, and are frequently identified in LUAD [28]. To evaluate the relevance of EGFR, KRAS and ALK mutations to these four subtypes, we characterized the patterns of the EGFR, KRAS and ALK mutations in LUAD data from TCGA. Subtype C3 and C4 showed a markedly lower proportion of EGFR mutations compared to C1 and C2 (p=9.54×10^−5^, Pearson’s chi-square test, Figure 4C). However, the KRAS mutation rate of subtype C4 was much lower than that of C1 and C3 (p=0.014, Pearson’s chi-square test, Figure 4C). Interestingly, the ALK mutation did not differ in our grouping, which indicates that it is not an immune-sensitive gene. (p=0.352, Pearson’s chi-square test, Figure 4C). We further analyzed the distribution of the number of all mutant genes in these four subtypes. Figure 4D shows that there were significant differences in the frequency of mutations among these groups (p=0, Pearson’s chi-square test). Genetic mutations were more likely to appear in C3, and less so in C1 and C4.

### Gene co-expression network analysis for the four subtypes

**Figure 5:**
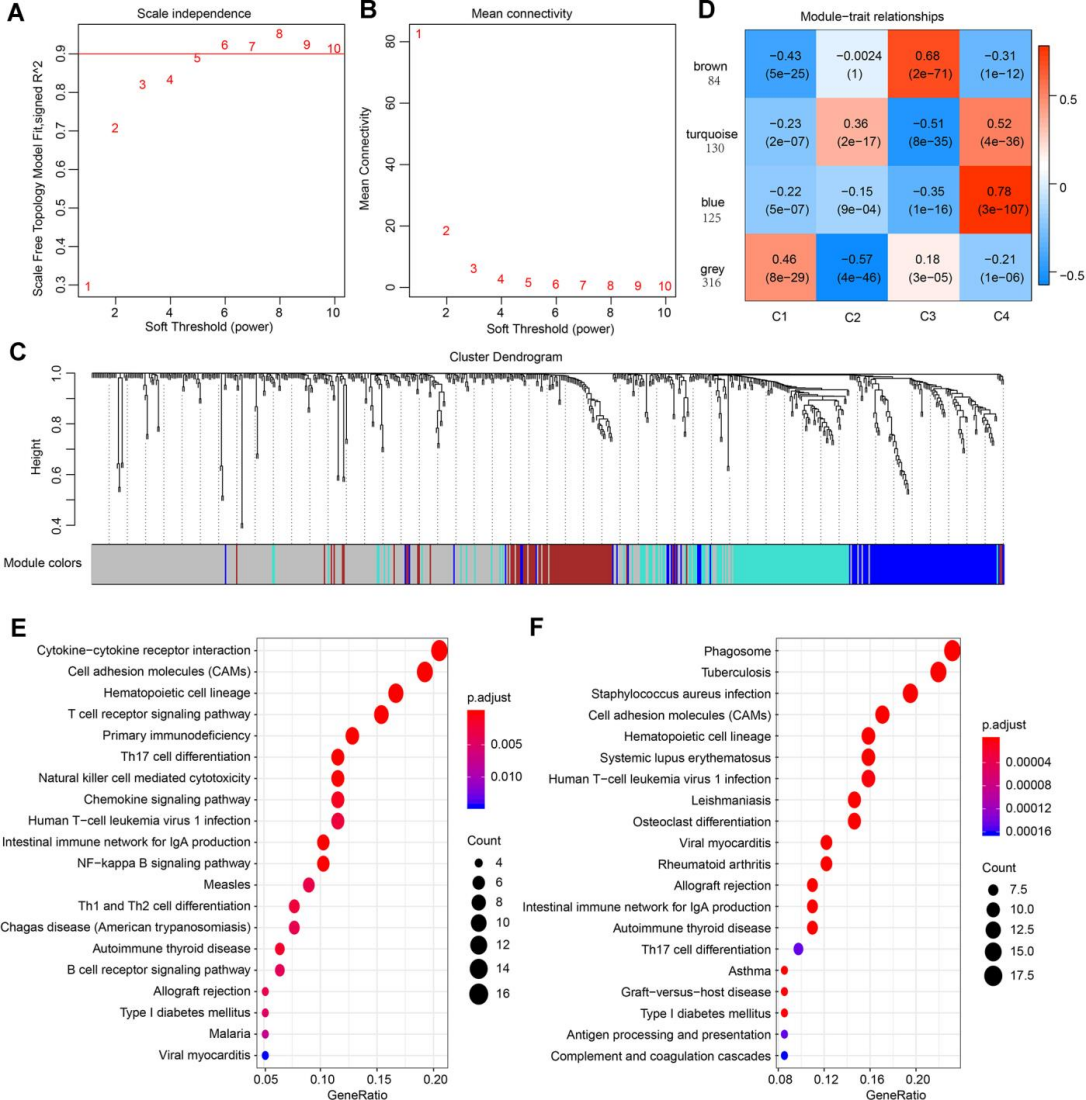
**Result of weighted gene correlation network analysis (WGCNA) analysis.** (**A**) The scale independence of WGCNA analysis and determination of parameter β of the adjacency function in the WGCNA algorithm. (**B**) The mean connectivity of WGCNA analysis. (**C**) Cluster results and trait heatmap of data samples. (**D**) Module-immune subtype weight correlations and corresponding P-values (in parenthesis). The left panel shows the four modules and the number of module member genes. (**E**) The top 20 pathways of genes in the blue module (ranked by FDR ≤ 0.05) in the KEGG database. (**F**) The top 20 pathways of genes in the turquoise module (ranked by FDR ≤ 0.05) in the KEGG database.

To classify genes with similar expression patterns into different modules for the four subtypes. Firstly, data of 655 differentially expressed immune-related genes of the four subtypes was grouped on the basis of similarity using the weighted gene co-expression network analysis (WGCNA) method [29]. In this analysis, a soft thresholding power of 5 was used and the best parameter β was 5 (Figure 5A, 5B). Then, we converted the expression matrix into an adjacency matrix, and the adjacency matrix into a topological matrix (TOM). Based on TOM, we used the average-linkage hierarchical clustering method to cluster genes according to their expression patterns across the subtypes. The dynamic shear method was employed to determine the gene modules, after which the eigengenes of each module were calculated. Subsequently, we clustered the modules and merged similar modules into one, then set height=0.25, deepSplit = 2, minModuleSize = 30. Four modules were acquired as shown in Figure 5C.

For better visualization, each gene cluster was assigned a specific color and a color code. A total of 655 genes were assigned into three co-expression modules (brown, turquoise, blue), while 316 genes that did not fit into other clusters were grouped into the fourth “grey” module. A key network was constructed using the Pearson correlation coefficients values of the four subtypes and modules (Figure 5D). Two modules were connected if they showed an absolute value of correlation > 0.45. Notably, the brown module was positively correlated with C3 (r=0.68, p=2e-71) and negatively correlated with C4 (r= -0.31, p=1e-12). In contrast, the blue module was strongly positively correlated with C4 (r=-0.78, p=4e-36) and negatively correlated with C3 (r=-0.35, p=1e-16). The turquoise module was also correlated with C4 (r=0.52, p=4e-36).

The KEGG enrichment analysis was performed to investigate the biological functions of the genes. Results showed that the blue module was enriched in 25 pathways, including immune-associated pathways such as primary immunodeficiency, the intestinal immune network for IgA production and T cell receptor signaling pathway. These observations were in agreement with previous reports [30, 31]. (Figure 5E). The genes in turquoise module were enriched in 32 pathways, and the top 20 pathways are shown in Figure 5F, including immune and inflammatory pathways such as Phagosome, Tuberculosis, and Inflammatory bowel disease (IBD). Interestingly, the genes in brown modules were not associated with KEGG pathways, indicating that the formation and pathogenesis of the subtype C3 are much more complicated and unknown.

### Validation of four molecular subtypes in the LUAD cohort

To validate the four molecular subtypes, we first selected the genes in the blue, turquoise, and brown modules closely related to C3 and C4 subtypes to calculate the correlation between genes and modules. Thirty-eight genes with correlation coefficients > 0.8 were identified and their expression profiles were extracted as training sets. The samples were clustered with the support vector machine, at a classification accuracy of 98.83%. Subsequently, GSE68465 data was downloaded from the GEO database and standardized into quantiles. A total of 462 samples were included, comprising 19 normal samples and 442 LUAD samples. After exclusion of 19 normal samples, 442 LUAD samples were analyzed. The expression profiles of genes in the blue, turquoise, and brown modules were extracted. The samples were subdivided into the model, of which 123, 72, 196, and 52 samples were predicted for subtype C1, C2, C3, C4, respectively. First, we analyzed the expression distribution of 13 immune metagenes in each subtype. As shown in Supplementary Figure 3, most metagenes were highly expressed in C4 and lowly expressed in C3, and this matched with the validation set. Consistent with TCGA cohort, subtype C4 in the GEO cohort was considered to be highly expressed among the immune signatures (Supplementary Figure 3A–3D). Most immune metagenes were highly expressed in C4 but lowly expressed in C3 (Supplementary Figure 3A). Analysis of immune microenvironmental factors suggested that the stromal score, immune score, and tumor purity were highest in subtype C4, but relatively lower in C3 (Supplementary Figure 3B). Besides, B lineage cells, NK cells, T cells, cytotoxic lymphocytes and myeloid dendritic cells were higher in C4 than in C3 (Supplementary Figure 3C). In the GEO cohort, subtype C4 had higher expression levels of checkpoint receptors PD1, CTLA-4, CD86 and CD80 and lower expression of VTCN1, compared with other subtypes (Supplementary Figure 3D). The expression value of CD276 and CD274, were not detected in the GEO dataset. In addition, significant survival differences were observed among the four subtypes in the GEO cohort (Supplementary Figure 3E, p=0.01973, log-rank). In particular, C4 was associated with enhanced immune microenvironment and showed the best prognosis. Further analysis of the relationship between the four subtypes in the GEO dataset and smoking history was conducted. As shown in Supplementary Figure 3F, the smoking degree differs among the subtypes (p=0.004, Pearson’s chi-square test). We further validate the four subtypes in GSE40419 dataset. Samples from GSE40419 were classified by the same method, of which 43, 40, 53, and 18 samples were predicted for subtype C1, C2, C3, C4, respectively. Consistently, most immune signatures were highly expressed in subtype C4 but lowly expressed in C3 (Supplementary Figure 4A–4D). Collectively, the findings from the GEO cohorts are in agreement with those from the TCGA cohort.

### CMap analysis for perturbagen signatures that reverse C3 immune subtype

**Figure 6:**
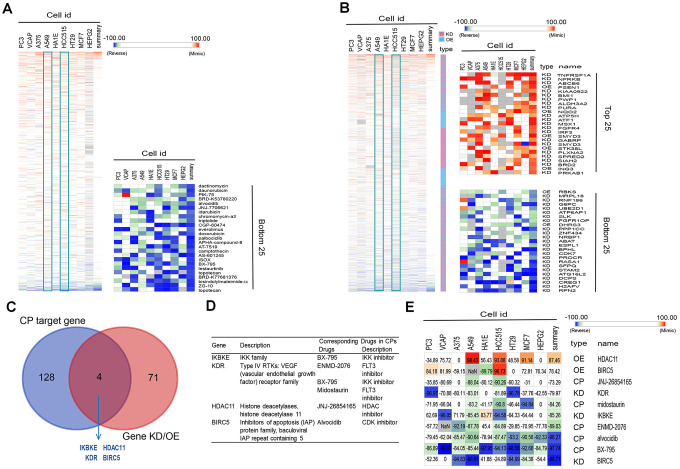
**Connectivity mapping for the gene signature in C3 immune subtype.** (**A**, **B**) Connections of C3-driven gene signature with the small molecule compounds (**A**) and gene knockdown/overexpression (**B**) were analyzed by querying the CLUE database. Connections were viewed as a heat map ranked by the summary connectivity score. (**C**) The venn diagram indicating the number of target genes of screened small molecule compounds (enrichment score<-80) and gene knockdown/overexpression (gene knockdown, enrichment score<-80; gene overexpression, enrichment score>80), and the overlap between each set of genes. (**D**) Descriptions of overlapped gene and their corresponding drugs from screened small molecule compounds. (**E**) Connections of C3-driven gene signature with screened small molecules and gene knockdown/overexpression were analyzed by querying the CLUE database. Connections were viewed as a heat map with each connectivity score in individual cell line. CP, compounds. KD, knockdown. OE, overexpression.

Among the four subtypes, we focused on patients with subtype C3. Their immunosuppressive status, accompanied by EGFR wild type, has been challenging to clinical treatment due to the lack of targets for tyrosine kinase inhibitor (TKI) and immunotherapy [6, 32]. To investigate potential drugs for this subtype, we applied computational drug repurposing strategies. Subsequently, we performed CMap analysis to identify new drugs that can reverse immune-suppressed status of subtype C3. Genes in the brown module that positively correlated with C3 (Supplementary Table 2) were recognized as up-regulated genes, and genes in blue module (Supplementary Table 2) were down-regulated genes. After being queried by the next-generation CMap database (CLUE, https://clue.io/), small molecule compounds (CPs) and genes knockdown or overexpress (KD/OE) with positive and negative scores and exhibiting similar or opposing gene expression signatures in group C3 are shown in Figure 6A, 6B. Our analysis was carried out using cell lines A549 and HCC515, two LUAD cell lines. We then selected CPs with enrichment scores of less than -80 in both adenocarcinoma cell lines as potentially capable of reversing the C3 aberrant gene expression (Supplementary Table 3). We next screened knockdown genes with scores lower than -80 and overexpressed genes with scores higher than 80 in the two cell lines as potential therapeutic targets against LUAD (Supplementary Table 4). This analysis identified four overlapping genes (IKBKE, KDR, HDAC11, BIRC5) among the known target genes of the selected CPs and screened genes (KD or OE). These candidates were confirmed as targets for C3 reversal and the CPs identified above (Figure 6C, 6D). Our analysis further revealed that, BX-795, ENMD-2076, midostaurin, JNJ-26854165 and alvocidib potently reverse the C3 subtype signature (Figure 6D, 6E). Interestingly, three drug candidates were identified for KDR. The connectivity scores for BX-795 and IKBKE knockdown were relatively similar in A549 and HCC515 cells.

### Validation of affinity of the candidate drugs by molecular docking analysis

**Figure 7:**
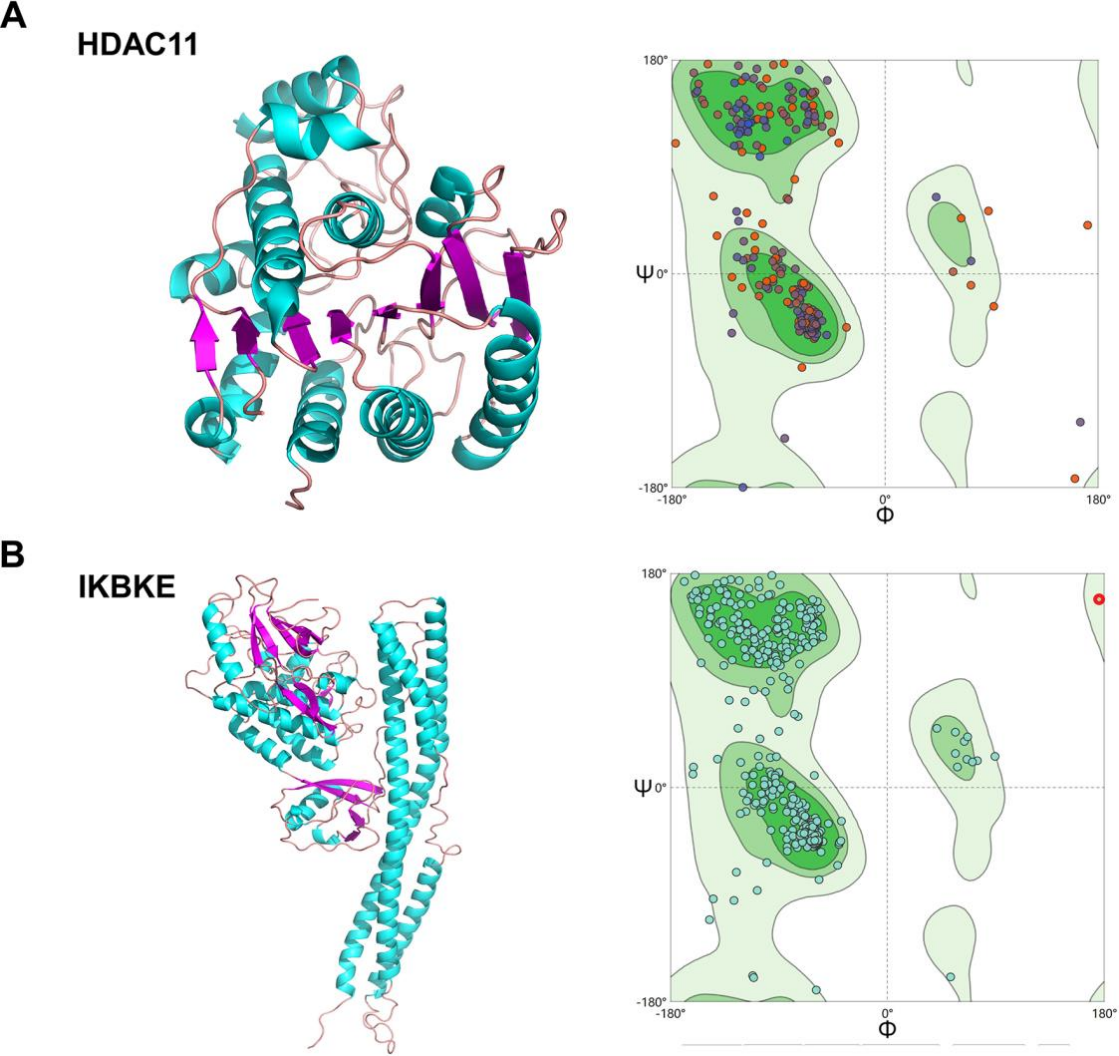
**Homologous modeling of HDAC11 and IKBKE protein structure.** (**A**) 3D structure of HDAC11 and IKBKE. (**B**) Ramachandran plot analysis.

**Figure 8:**
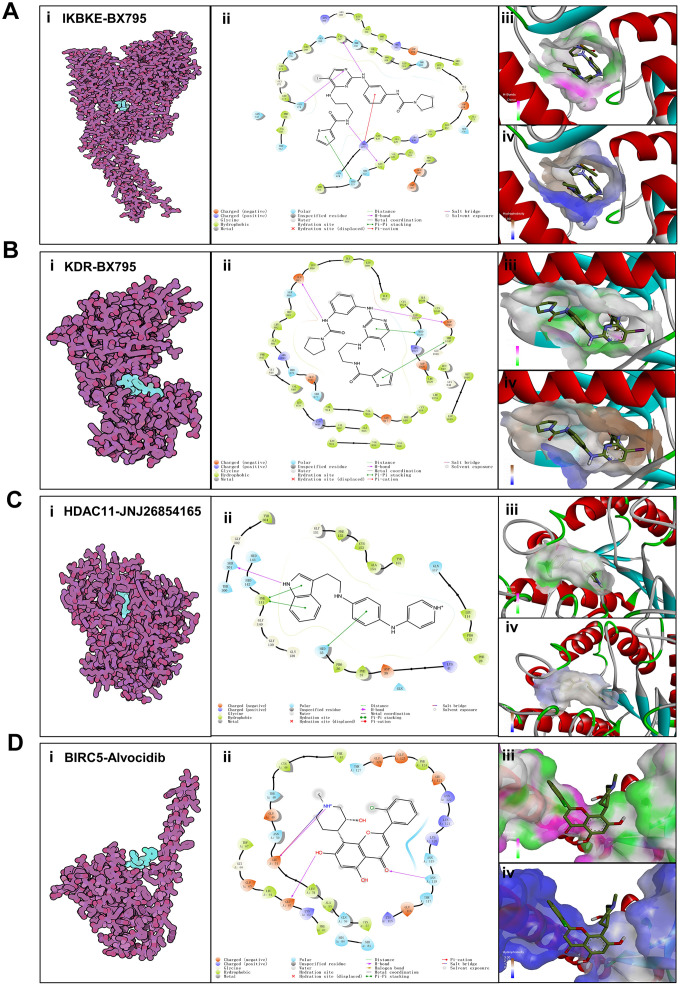
**Binding mode of screened drugs to their targets by molecular docking.** (**A**) Binding mode of BX795 to IKBKE. (**B**) Binding mode of BX795 to KDR. (**C**) Binding mode of JNJ26854165 to HDAC11. (**D**) Binding mode of Alvocidib to BIRC5. (**i**), Cartoon representation, overlay of the crystal structures of small molecule compounds and their targets were illustrated by Molecule of the Month feature. (**ii**), 2D interactions of compounds and their targets. (**iii**, **iv**) Three-dimensional structures of the binding pockets were showed by PyMOL software. (**iii**), Coloring is from carmine (for strong H-bonds) to green (for poor H-bonds). (**iv**), Coloring is from magenta (for strong hydrophobic regions) to blue (for poor hydrophobic regions).

To evaluate the affinity of the candidate drugs for their targets, we performed molecular docking analysis. First, 3D models of HDAC11 and IKBKE protein structure were predicted using the template-based homology modeling approach. Consequently, 6HSK-A and 4IM0-A (PDB structures) were identified as ideal templates for modeling as they demonstrated high sequence similarity (32% and 44%) [33]. Ramachandran plot analysis demonstrated existence of 92.5% of all residues in the allowed regions for HDAC11 and 94.7% for IKBKE, highlighting the accuracy of the predicted structures (Figure 7). The binding poses and interactions of five drug candidates with four protein were obtained with Autodock Vina v.1.1.2 and binding energy for each interaction was generated (Figure 8, Supplementary Figure 5 and Table 1). Results showed that each drug candidates bound to its protein targets through visible hydrogen bonds and strong electrostatic interactions. Moreover, the hydrophobic pockets of each targets were occupied successfully by the five candidate drugs. For KDR, two candidates, JNJ-26854165 and BX-795 had low binding energy of -9.7 and -9.3 kcal/mol, indicating highly stable binding (Table 1).

**Table 1:** Binding Energy for targets with their drugs.

**Target**	**Drug**	**Binding Energy (kcal/mol)**
IKBKE	BX-975	-8.3
KDR	ENMD-2076	-7.8
KDR	BX-795	-9.3
KDR	Midostaurin	-0.8
HDAC11	JNJ-26854165	-8.9
BIRC5	Alvocidib	-5.4

## DISCUSSION

In recent years, increasing studies identifying and stratifying the immune characteristics of patients with LUAD have been reported [34–37]. Yet, most of them focused solely on the clinical relevance such as survival and prognosis, and have not been translated into routine clinical practice. This calls for a further exploration and summarization of the LUAD microenvironment to expose the molecular events underlying tumor cell–immunocyte interactions, in particular, the relevance study of drug development.

In this study, we report a model for the practice of personalized immunotherapy, which is to couple patient grouping and exploration of novel therapeutic candidates. The four LUAD immune subtypes grouped on the basis of immune related gene expression profiles were associated with distinct molecular characteristics and clinical outcomes. Subtype C4 showed high levels of infiltration and expression of PD1, CTLA-4 and their receptors, meeting the criteria for classification as “hot” tumors. Of the four subgroups, patients belonging to subgroup C3 exhibited poor lymphocyte infiltration and the lowest expression of immune checkpoint proteins meeting the criteria for classification as “cold” tumors. Additionally, subtype C3 showed significantly lower median age at diagnosis, the highest proportion of male patients, smokers and the highest frequency of mutant genes. Interestingly, although C3 group had the highest frequency of gene mutation among the four subtypes, it harbored much fewer therapeutically important EGFR alterations, indicating that patients with this subtype can hardly benefit from immunotherapy or tyrosine kinase inhibitor (TKI) alone.

Nowadays, the combination of priming therapy to enhance T cell responses along with the removal of inhibitory signals (and/or the supply of co-stimulatory signals) has been proposed to convert “old” tumors into “hot” tumors and overcome the lack of pre-existing immune responses [7]. However, the development of novel drugs is costly and time-consuming. Consequently, drug repurposing, where existing medication are utilized for the treatment of conditions other than their original targets has emerged as a potential solution to these challenges.

A critical assumption of CMap analyses is that a drug that induces changes in gene expression that are opposite to those caused by a disease may have potential therapeutic benefits against the disease. Therefore, the outputs from inputting the blue and brown modules into CMap are potential targets and drugs that can reverse the cold tumor status of the C3 subgroup. Here, we identified five drugs against four targets associated with the C3 status. IKBKE has been described to impact on inflammatory and metabolic diseases as well as on cell proliferation and transformation [38]. BIRC5 (baculoviral IAP repeat containing 5) is overexpressed in various tumors and associated with poor cancer survival [39]. KDR (also called VEGFR2) is a key modulator of angiogenesis and its overexpression is frequently associated with poorer prognoses in lung cancer patients [40]. It is notable that inhibition of KDR alleviates hypoxia and remodels the immunosuppressive tumor microenvironment [41]. It has also been reported that HDAC11 inhibition might regulate immune activation by increasing type I interferon signaling [42]. These indicates that the inhibition of these four targets has potential dual functions to correct aberrant immune microenvironment also halt tumorigenesis at the same time.

Of all drug candidates, midostaurin needs special attention because it has gained approval by the FDA for the treatment of acute myeloid leukemia (AML) [43]. Interestingly, midostaurin has been found to displayed potent antiproliferative activity in several lung cell lines [44]. Another concern is BX-795, a known multi-target kinase inhibitor [45, 46]. Researches in recent years found that it exhibited inhibitory activity against virus infection and various cancer [47–49]. In this work, we found that BX795 can inhibit IKBKE and KDR at the same time correct aberrant gene expression in the C3 subgroup. The only oral drug among all candidates is ENMD-2076. This is a multi-target kinase inhibitor with antitumor activities against breast cancer, melanoma, colorectal cancer [50–53]. Alvocidib can be used as a metastasis inhibitor and an apoptosis inducer in KRAS mutant population especially since KRAS mutation rate of C3 group was high [54]. Over all, all identified compounds including JNJ-26854165 [55] have previously shown the potential to inhibit a variety of tumors, of which midostaurin has been clinically approved for the treatment of hematological diseases. Furthermore, their steady binding affinity against the targets was verified through molecular docking analysis at a molecular level, thus warranting further investigation to validate.

This strategy can also be used to immunotype other tumor patients and to screen for potential personalized drugs. Recently, immunotherapy, especially immune checkpoint blockade (ICB, e.g. anti-PD1/PD-L1 antibodies), has been used to treat multiple cancers, including NSCLC, melanoma, renal cell cancer, colorectal cancer, recurrent head and neck cancer (squamous cell), urothelial carcinoma, gastric cancer cervical cancer [56]. However, response to current immunotherapies and survival benefits are often seen in a subset of patients. The key to solving this problem lies in determining the individual's ability to respond to immunotherapy and to design a rational, individualized immunotherapy combined strategy. Therefore, to enhance and improve the efficacy of current immunotherapy, a better understanding of tumor immune microenvironment is required. As shown in this paper, in other tumors such as melanoma, we can also use unsupervised consensus cluster analysis, which relies on the expression profiles of immune-related genes, to reveal the immune landscape and characteristics within the tumor. Furthermore, based on immunophenotypic features, WGCNA analysis can be applied to construct co-expression networks and identify hub genes. After drug repurposing, the identified potential therapeutic candidates may help facilitate personalized immunotherapy for patients with different molecular subtypes. In conclusion, it is evident that this method can be applied to other tumor types in which therapeutic response is dependent on the immune microenvironment.

In summary, this study highlights the potential of coupling patient stratification with drug repurposing strategy as an alternative means for developing personalized immunotherapy.

## MATERIALS AND METHODS

### Sample datasets and clinical profiles

The clinical data and gene expression profiles of 513 LUAD data obtained from The Cancer Genome Atlas (TCGA) were used to analyze the immune microenvironment and molecular subtype of LUAD [57]. Data on overall survival (OS) (distant or locoregional recurrence after surgical treatment) was extracted from the TCGA cohort. The two LUAD expression datasets, including GSE68465 and GSE40419, as well as the corresponding clinical information in Gene Expression Omnibus (GEO) [58] were included to validate our results. The OS data were extracted from the GEO cohort.

### Processing of gene expression data

For the TCGA cohort, data of the fragments per kilobase of gene per million fragments (FPKM) was derived from the TCGA data portal. Next, the expression values of FPKM were converted to transcripts Per Kilobase of exon model per Million mapped reads (TPM) for subsequent analysis. The genes were annotated using the Ensembl database. The clinical information and normalized expression data of the GEO cohort was obtained from the Gene Expression Omnibus (GEO) (GSE68465). Probe annotations of BeadChips were derived from the GEO database. The expression data of the two cohorts were mapped using the Entrez Gene.

### Characterization of molecular subtypes of LUAD using immune genes

We analyzed whether the expression profile of global immune-related genes in the TCGA cohort could distinguish the LUAD subtypes. The expression data of immune-associated genes was derived from the Immunology Database and Analysis Portal (ImmPort) database (https://immport.niaid.nih.gov). The immune-related genes with expression level > 0 (FPKM>0) in more than 30% of samples were included, resulting in 790 genes selected for subsequent Consensus Cluster Plus analysis. The similarity distance between samples was calculated by the Euclidean distance metric. The samples were clustered using the k-means clustering algorithm, with 1000 iterations by sampling 80% of the samples in each iteration. The cluster numbers varied from 2 to 8, and the optimal partition was determined by evaluating the consensus cumulative distribution function (CDF) [59]. The pair comparisons between the identified subtypes were determined by SigClust analysis. Bonferroni correction was applied for multiple testing. The Kolmogorov-Smirnov test was used to identify highly expressed genes among the subtypes. The false discovery rate (FDR) was determined by the Benjamini-Hochberg method. FDR<0.05 was set as the threshold. In each subtype, the top 100 upregulated genes were employed to distinguish among the immune molecular subtypes.

### Immune signature analysis in LUAD molecular subtypes

Thirteen immune metagenes corresponding to various immune cells and related immune functions were derived from a previous publication [27]. The expression scores of micro-environmental factors (tumor, immune, and stromal purity) were obtained using the ESTIMATE algorithm [60]. The association among the tumor samples and six tumor-infiltrating lymphocytes including B, and dendritic cells, neutrophils, CD8+ T, macrophages, CD4+ T, was analyzed using TIMER (https://cistrome.shinyapps.io/timer). The Microenvironment Cell Populations (MCP)-counter method developed by Etienne Becht et al. was used to validate the immune profiles [61]. MCP-counter was used to estimate the inter-sample relative abundance of immune infiltrates based on gene expression profiles. The R package “MCPcounter” was utilized to calculate the MCP-counter scores. The expression score of immune signatures in each tumor sample was calculated using the log2 transformed and median-centered FPKM expression values and then visualized by heatmap. The immune signature and expression level of checkpoint genes were also analyzed in all molecular subtypes. Different LUAD subtypes were compared by Analysis of Variance (ANOVA) test. Multiple testing was performed by Bonferroni correction.

### Analysis of mutations in each subtype

The EGFR-mutant, KRAS-mutant and ALK mutant data were extracted from the SNP dataset in TCGA after processing with MuTect method (http://www.broadinstitute.org/cancer/cga/mutect) [62]. The frequency of mutations was assessed by calculating the number of variants annotated by ANNOVAR [63, 64].

### Weighted Gene Co-expression Network Analysis (WGCNA) Analysis

The WGCNA package in R software was employed to execute WGCNA analysis. Initially, Pearson correlation coefficients (ranging from − 1 to 1) were used to calculate the co-expression of all gene pairs. Due to the small sample size enrolled in the present study, Pearson correlations measuring linear relationships were chosen to minimize overfitting. To convert the correlation coefficients into a weighted adjacency matrix (values ranging from 0 to 1), we raised the co-expression similarity to a power β = 5. The adjacency matrix enables the determination of the strengths of connection between two nodes. The matrix is therefore used to establish a topological overlap matrix (TOM) which addresses the topological similarity factor. Here, we used the TOM to determine the corresponding dissimilarity (1-TOM) for cluster formation. Genes with clear expression patterns were classified into modules using the average linkage hierarchical clustering in concert with TOM-based dissimilarity. Specifically, gene modules (clusters of densely interconnected genes in terms of co-expression) were detected using the dynamic tree-cutting algorithm (deep split = 2, minimum number of genes per module = 30, cut height = 0.25). Unassigned genes were represented by gray color, while all other modules were assigned different colors in a random manner. Determination of modules highly correlated with subtypes was achieved using the module eigengenes (MEs). All analyses were carried out using the WGCNA package.

### Functional group analysis

ClusterProfiler software 3.6.0 was employed for KEGG pathway enrichment analysis of the genes in each module and subtype. The R package of this software helps to determine the biological functions of gene clusters and to compare several gene clusters [65].

### Connectivity map analysis

The next generation Connectivity Map (CMap, https://clue.io/) is a database that catalogs gene expression profiles of various human cell lines upon exposure to various small molecule compounds and genetic perturbations [66, 67]. To find perturbagens that reverse the immune-suppressed status of subtype C3, the genes listed in Supplementary Table 2 were inputted as query into the CLUE database and results downloaded from the CMap database. Compounds (CPs) with potential to reverse the C3 phenotype were further screened by filtering for an enrichment score of <−80 in both A549 and HCC515 cell lines. Gene knockdown (KD) with enrichment scores below -80 and overexpressed genes (OE) with scores above 80 in both A549 and HCC515 were also identified. Overlapping genes between target genes of CPs and genes KD or OE that perturb the C3 signature were determined by Venn diagram analysis. The overlapping genes were considered potential drug targets for C3 group. We hypothesized that these candidate genes can be pharmacologically targeted with the identified CPs with scores of <−80. Connections of C3 driven gene signature to CPs or gene KD/OE were obtained from the results and presented in the form of a heat map.

### Homologous modeling

To analyze the binding affinities and modes of interaction between the CPs and their targets, we used an in silico protein-ligand docking software (AutodockVina 1.1.2) [68]. To date, there is no complete crystal structure of HDAC11 and IKBKE, so their amino acids sequences were analyzed by EXpasy (http://swissmodel.expasy.org/) [69] Ramachandran plots were used to assess stereo-chemical quality [70]. The parameters were set to default.

### Molecular docking

The molecular structures of ENMD-2076, BX-795, JNJ-26854165, midostaurin and alvocidib were retrieved from PubChem Compound (https://pubchem.ncbi.nlm.nih.gov/) [71]. The 3D coordinates of KDR (PDB ID, 5EW3; resolution, 2.5 Å) and BIRC5(PDB ID, 4AOI; resolution, 1.9Å) were downloaded from the PDB (http://www.rcsb.org/pdb/home/home.do). For docking analysis, all protein and molecular files were converted into PDBQT format with all water molecules excluded and polar hydrogen atoms were added using MGLTools (version 1.5.6). The grid box was centered to cover the domain of each protein and to accommodate free molecular movement. The grid box was set to 30 Å × 30 Å × 30 Å, and grid point distance was 0.05nm. Molecular docking studies were performed by Autodock Vina 1.1.2 (http://autodock.scripps.edu/) and Pymol software 2.3 (DeLano Scientific, Portland, USA) was used for model visualization.

### Statistical methods

Fisher’s exact test or chi-square test was used to evaluate the correlation between molecular subtypes and conventional clinical variables. Benjamini-Hochberg’s FDR was used for corrected multiple testing. The log-rank tests and Kaplan-Meier curves were used to calculate the OS rates for each molecular subtype. These statistics were two-sided and were performed using R software.

### Availability of supporting data

The datasets analyzed during the current study are available in the Genomic Data Commons (GDC, https://gdc.cancer.gov/access-data/gdc-data-portal) and Gene Expression Omnibus (GEO, https://www.ncbi.nlm.nih.gov/geo/) repositories.

## Supplementary Material

Supplementary Figures

Supplementary Table 1

Supplementary Table 2

Supplementary Table 3

Supplementary Table 4
