# Supplementary material for: Coupled immune stratification and identification of therapeutic candidates in patients with lung adenocarcinoma
Source: Aging (Albany NY). 2020 Aug 27;12(16):16514–38. doi: 10.18632/aging.103775 (PMC7485744; doi:10.18632/aging.103775)
Supplement: Supplementary Figures [file aging-12-103775-s005..pdf]

SUPPLEMENTARY FIGURES

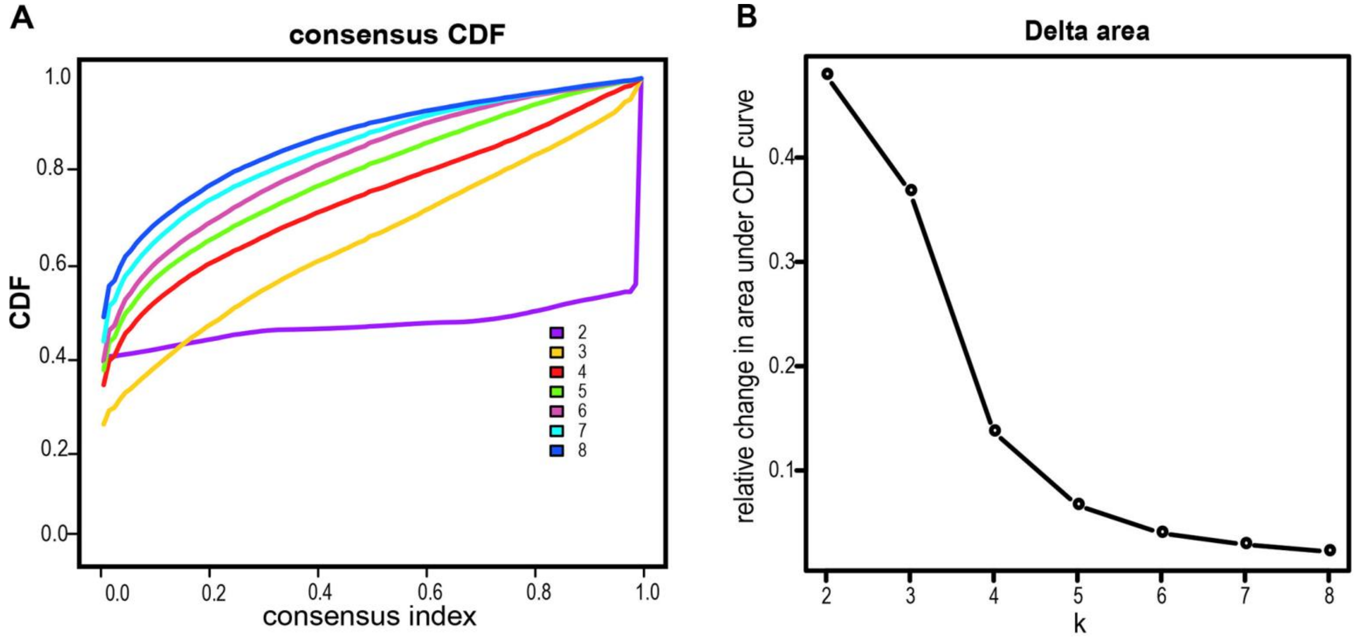

Supplementary Figure 1. The cumulative distribution function (CDF) curves. (**A**) The CDF curves in unsupervised consensus cluster analysis. The cluster numbers (k = 2, 3, 4, 5, 6, 7, 8) and their corresponding consensus scores. (**B**) Relative changes of the area under CDF curves. The number of clusters are shown on the x-axis. The y-axis indicates the proportion of areas under CDF curve.

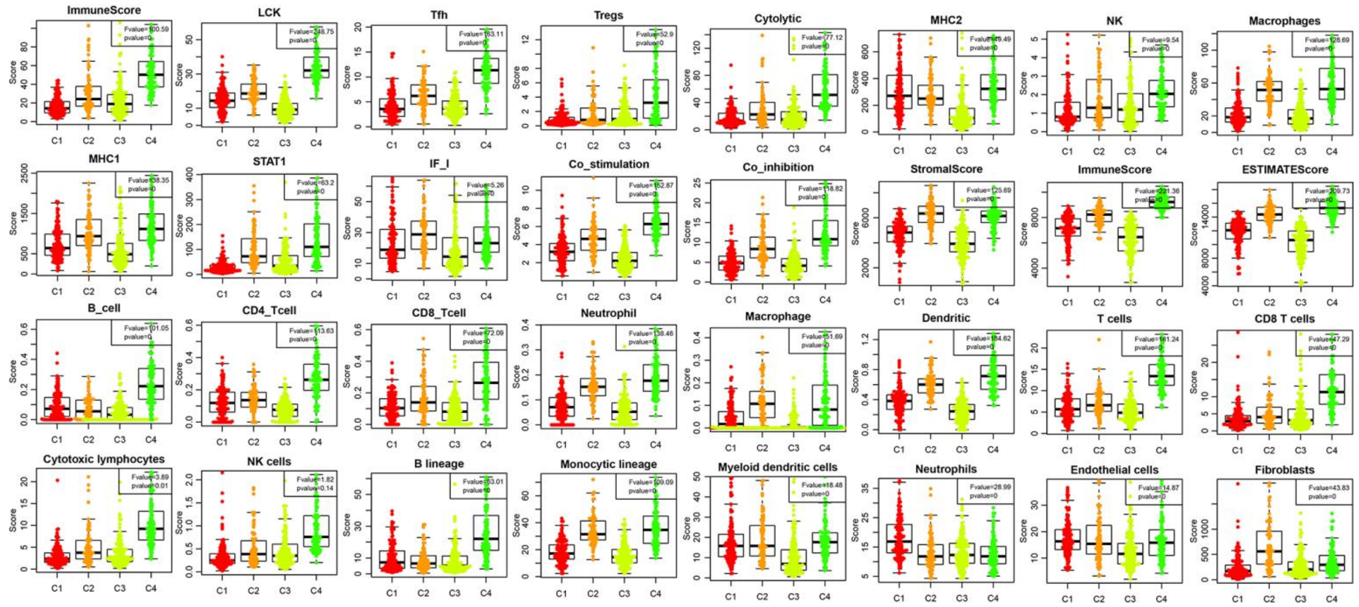

Supplementary Figure 2. Boxplots showing the gene expression scores of immune profiles of the four subtypes. Boxplots show 5%, 25%, 50%, 75%, and 95%, respectively.

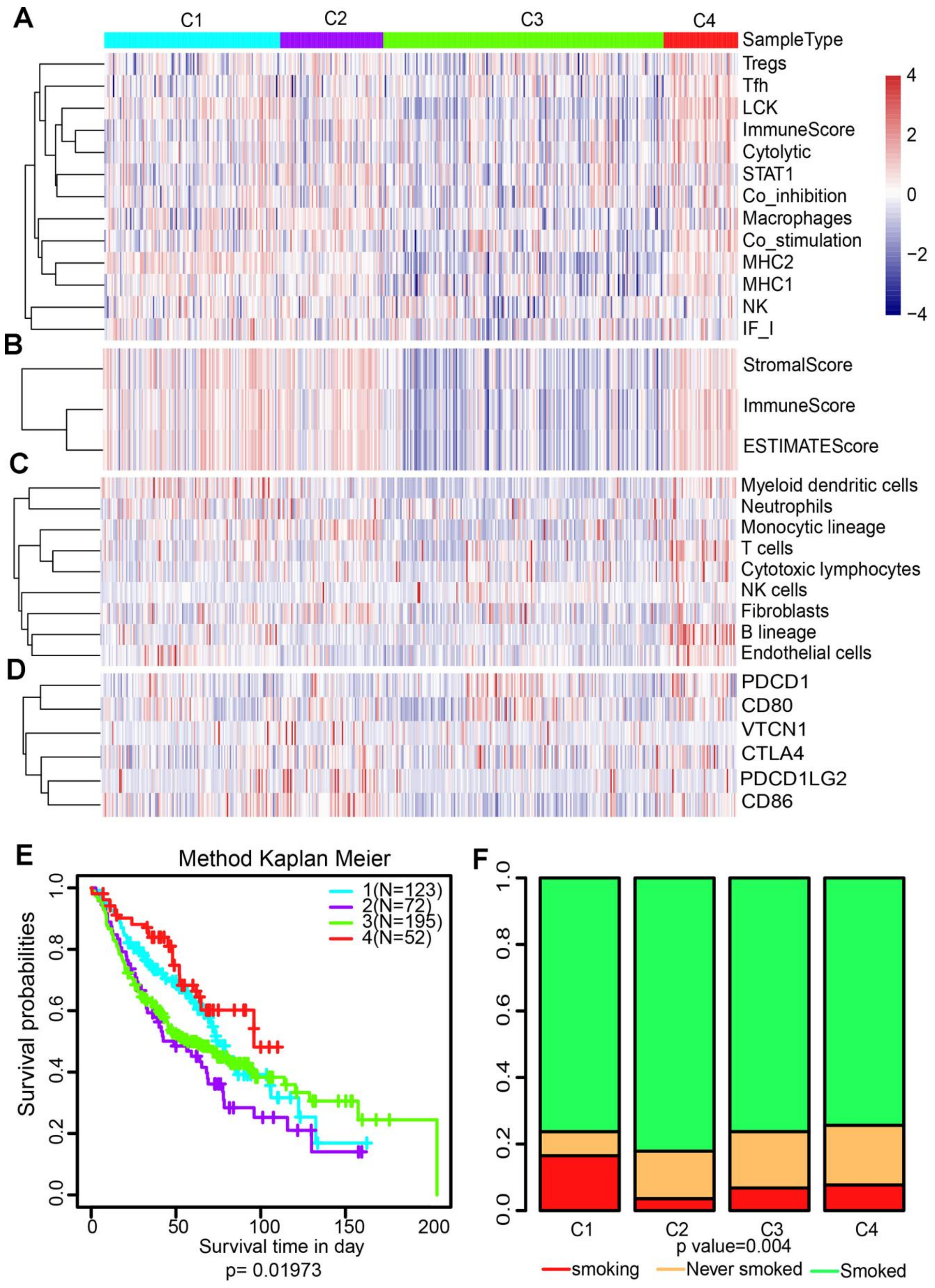

Supplementary Figure 3. Validation of the four immune-associated subtypes in the GEO LUAD cohort. (**A**) The expression levels of 13 immune metagenes. (**B**) The expression levels of genes included in the ESTIMATE algorithm for determination of stromal and immune gene signatures. (**C**) The expression scores of genes related to 10 groups of immune cells. (**D**) The expression scores of checkpoint molecules among the four subtypes in the GEO LUAD cohort. (**E**) Kaplan–Meier curves showing the overall survival (OS) of the four subtypes (log-rank test). (**F**) Distribution of smoking status among the four subtypes.

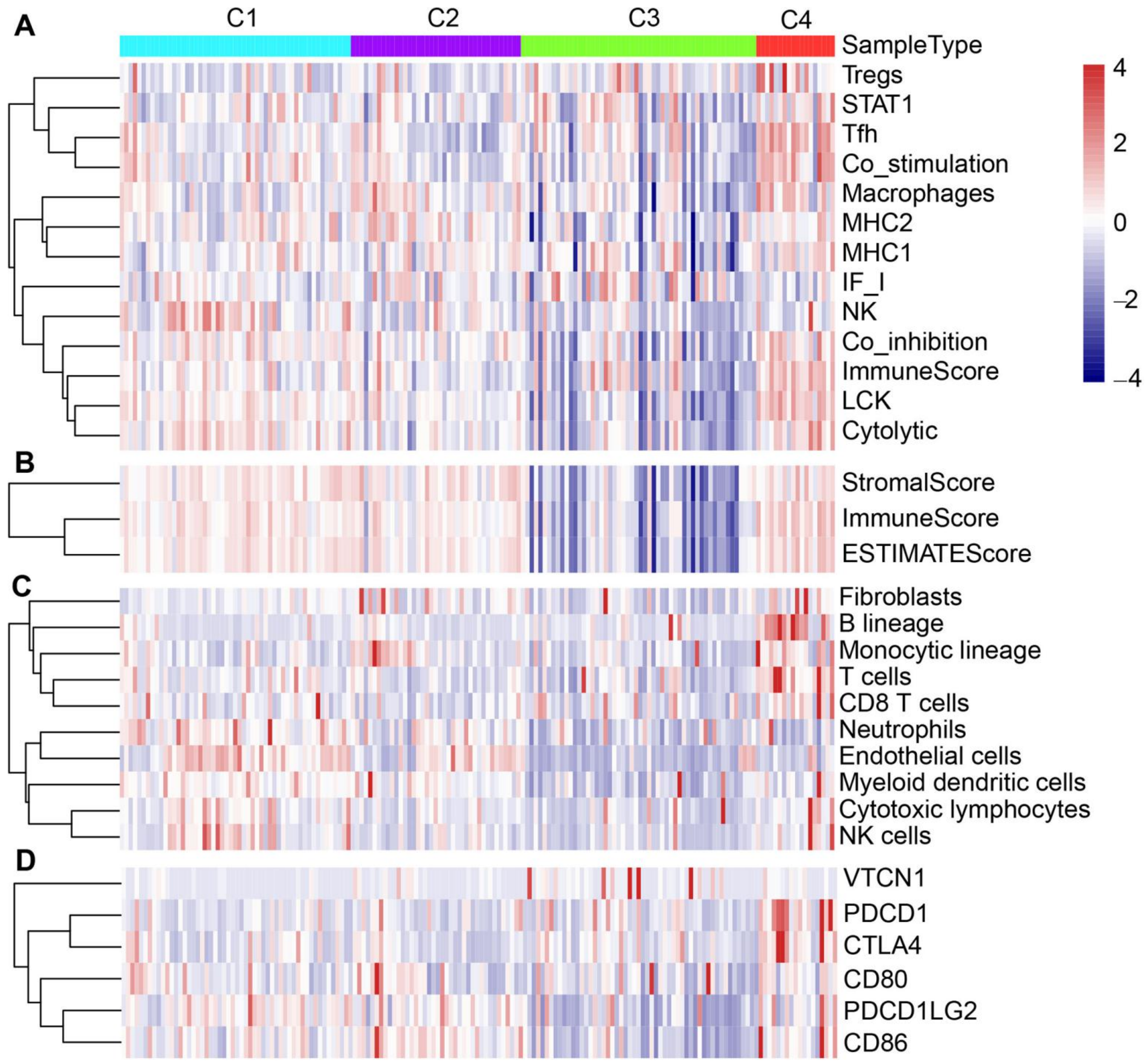

Supplementary Figure 4. Validation of the four immune-associated subtypes in the GSE40419 dataset. (**A**) The expression levels of 13 immune metagenes. (**B**) The expression levels of genes included in the ESTIMATE algorithm for determination of stromal and immune gene signatures. (**C**) The expression scores of genes related to 10 groups of immune cells. (**D**) The expression scores of checkpoint molecules among the four subtypes.

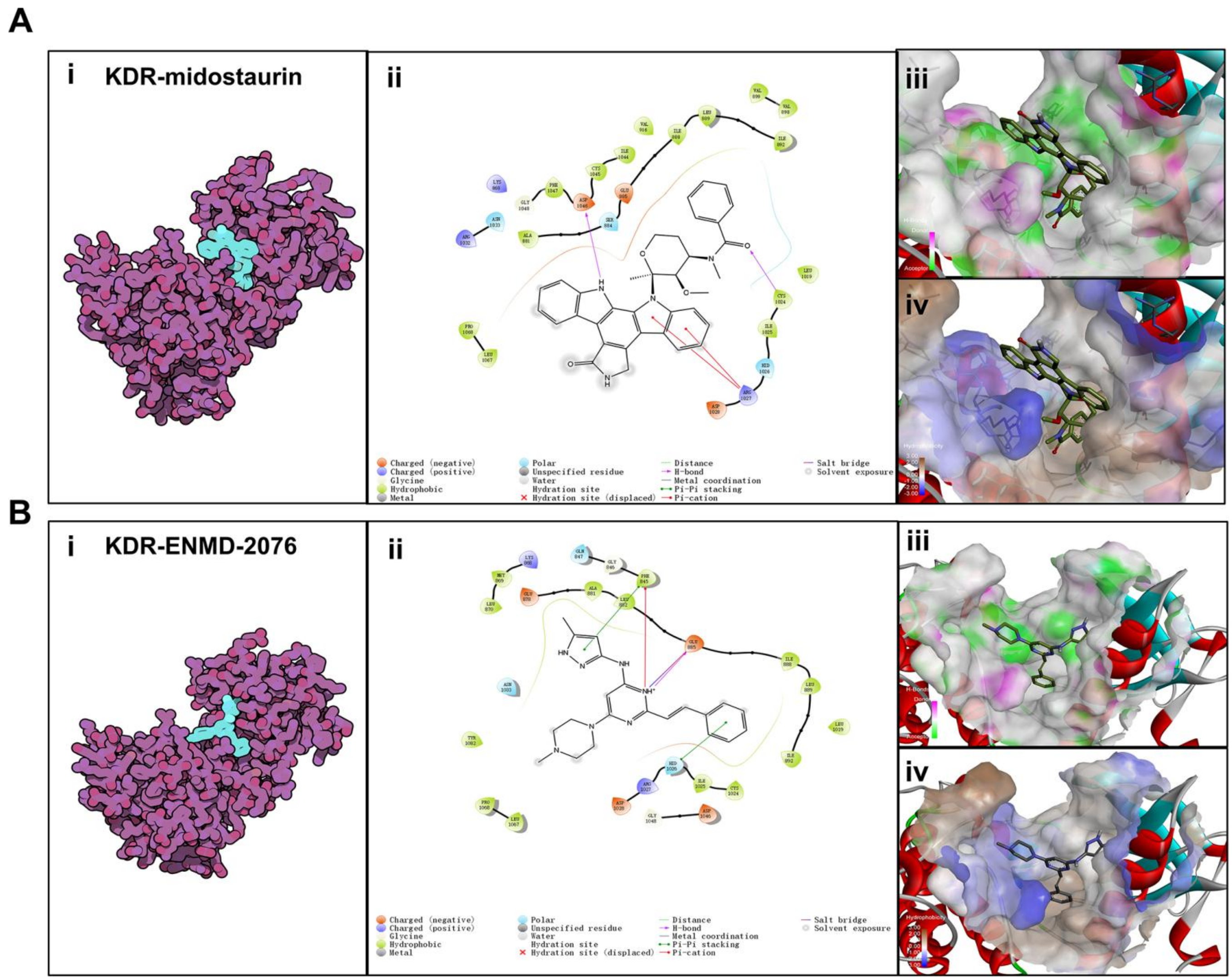

Supplementary Figure 5. Binding mode of screened drugs to KDR protein by molecular docking. (**A**) Binding mode of midostaurin to KDR. (**B**) Binding mode of ENMD-2076 to KDR. (**i**), Cartoon representation, overlay of the crystal structures of small molecule compounds and their targets were illustrated by Molecule of the Month feature. (**ii**), 2D interactions of compounds and their targets. (**iii**, **iv**) Three-dimensional structures of the binding pockets were showed by PyMOL software. (**iii**), Coloring is from carmine (for strong H-bonds) to green (for poor H-bonds). (**iv**), Coloring is from magenta (for strong hydrophobic regions) to blue (for poor hydrophobic regions).
