# Supplementary material for: Coupled immune stratification and identification of therapeutic candidates in patients with lung adenocarcinoma
Source: Aging (Albany NY). 2020 Aug 27;12(16):16514–38. doi: 10.18632/aging.103775 (PMC7485744; doi:10.18632/aging.103775)
Supplement: Supplementary Table 1 [file aging-12-103775-s004..pdf]

SUPPLEMENTARY TABLE

Supplementary Table 1. The clustering significance between the four subtypes.

P-values	C1	C2	C3	C4
C1	1	0.140006283	0.062373838	0.00755249
C2	0.140006283	1	0.19246147	0.123329892
C3	0.062373838	0.19246147	1	0.000127256
C4	0.00755249	0.123329892	0.000127256	1
